# Antimicrobial breakpoint estimation accounting for variability in pharmacokinetics

**DOI:** 10.1186/1742-4682-6-10

**Published:** 2009-06-26

**Authors:** Goue Denis Gohore Bi, Jun Li, Fahima Nekka

**Affiliations:** 1 Faculté de Pharmacie, Université de Montréal, Montréal, Québec, Canada; 2 Centre de Recherche Mathématiques, Université de Montréal, Montréal, Québec, Canada; 3 Pharsight, Montréal, Québec, Canada; 4 Groupe de recherche universitaire sur le médicament (GRUM), Université de Montréal, Montréal, Québec, Canada

## Abstract

**Background:**

Pharmacokinetic and pharmacodynamic (PK/PD) indices are increasingly being used in the microbiological field to assess the efficacy of a dosing regimen. In contrast to methods using MIC, PK/PD-based methods reflect *in vivo *conditions and are more predictive of efficacy. Unfortunately, they entail the use of one PK-derived value such as AUC or Cmax and may thus lead to biased efficiency information when the variability is large. The aim of the present work was to evaluate the efficacy of a treatment by adjusting classical breakpoint estimation methods to the situation of variable PK profiles.

**Methods and results:**

We propose a logical generalisation of the usual AUC methods by introducing the concept of "efficiency" for a PK profile, which involves the efficacy function as a weight. We formulated these methods for both classes of concentration- and time-dependent antibiotics. Using drug models and *in silico *approaches, we provide a theoretical basis for characterizing the efficiency of a PK profile under *in vivo *conditions. We also used the particular case of variable drug intake to assess the effect of the variable PK profiles generated and to analyse the implications for breakpoint estimation.

**Conclusion:**

Compared to traditional methods, our weighted AUC approach gives a more powerful PK/PD link and reveals, through examples, interesting issues about the uniqueness of therapeutic outcome indices and antibiotic resistance problems.

## Background

Antimicrobial efficiency and resistance have become a global public health issue and a real challenge for microbiologists, pharmaceutical companies, physicians and other members of the health community. Inadequate use of antibiotics promotes the selection of bacteria with decreased susceptibility. The search for new drugs to treat infectious diseases, the traditional approach to overcoming antibiotic resistance, is growing more challenging because multiple-resistance is becoming more prevalent among bacteria, and new targets for antimicrobial antibacterial action remain to be discovered [1-3]. The development of new antimicrobial antibiotics is a long, costly process, which takes a poor second place to the development of more lucrative drugs for an aging population. Therefore, improving the current use of antibiotics is central to preserving their long-term effectiveness in humans and animals. For public health officials, susceptibility testing data are crucial for the surveillance and control of emerging resistance. To collect these data, several susceptibility testing methods including dilution, disk diffusion and automated instrument system methods are currently in routine laboratory use [1-3]. To interpret the susceptibility test results, the breakpoint, a discriminating concentration, has been used to define isolates as susceptible, intermediate or resistant [4-6]. For obvious reasons of drug efficacy and antibiotic resistance problems, estimation of breakpoints has become a necessary step in modern microbiology laboratory practice. Breakpoints are estimated in a variety of ways, the most widely used being the minimal inhibitory concentration (MIC), which is the lowest concentration that completely inhibits microbial growth [1,3,7]. Although the MIC is considered the gold standard for breakpoint assessment, its main drawback lies in its *in vitro *basis, with no drug disposition information being included. In fact, MIC is a threshold value while antibacterial efficacy is a complex consequence of dynamic concentration- and time-dependent processes. In recent decades, these limitations have led professional groups to make intensive efforts to review pharmacokinetic and clinical data and establish suitable drug breakpoints under *in vivo *conditions. One of latest tendencies is to integrate PK/PD indices in order to understand the relevance of drug dose and schedule to efficacy [4,8-18]. The breakpoints obtained, generally called pharmacokinetic/pharmacodynamic (PK/PD) breakpoints, refer to the antibacterial concentrations calculated from the knowledge of a PD parameter and the dimension of that parameter for predicting efficacy *in vivo *[19]. The specific PK/PD indices correlating with bacteriological efficacy mostly depend on the nature of drug action in bacterial killing, which may be either concentration-dependent or time-dependent [20]. There has been a great increase in interest in the use of PK/PD studies to estimate drug efficacy since the foundation of the International Society for Anti-infective Pharmacology (ISAP) in 1991 [20]. Whilst these methods are more realistic as they are adapted to *in vivo *conditions, they still are empirically based, lacking a theoretical or mechanistic basis. Most importantly, the role of variability between individuals and from other potential sources cannot be explained in a definite way. This situation has clearly restricted the further development of these approaches. Because of this experimental limitation and the complexity of the problem, there is a need to develop new methodologies for drug evaluation. In this work, we provide a theoretical basis for characterizing the "efficiency" of a PK profile under *in vivo *conditions, which will then be supported by *in silico *approaches adopted for the two classes of concentration- and time-dependent antibiotic drugs. Using this approach, breakpoints can be explained and estimated within the context of standard PK/PD analysis.

Two patterns of antibiotic performance are often used to regroup antibacterial agents according to their bacterial controlling activities [21-24]. The first pattern, characterized by concentration-dependence, refers to drugs that have bacterial killing capacities covering a wide range of concentrations and effects proportional to concentration. The second one, known as time-dependent pattern is mainly exhibited by drugs with a saturated killing capacity directly linked to exposure time. This class also includes antibiotics of which the action is predominantly bacteriostatic (inhibit bacterial growth). Although there are many reported classes of antimicrobial agents, such agents generally fall into one of these two major patterns [23,25]. Published work about these two groups of drugs shows that the research community is allocating increasing interest to this important topic. Of particular note is the increasing popularity of PK/PD-based methods for predicting and measuring the therapeutic outcomes of these two groups of drugs [20,26]. Table 1 summarises the evolution of research on antimicrobial agents in terms of their activity patterns and the progress in PK/PD-based methods.

**Table 1:** Report on the antibacterial agents for different activity patterns and methods*

	**Year**				
**Types or Methods**	1970–1980	1981–1990	1991–2000	2001–2008	**Drugs or parameters**
Time-dependent	15	60	104	228	Beta-lactams Macrolides

Concentration-dependent	10	80	225	315	Aminoglycosides Fluoroquinolone

PK/PD-based methods	0	20	141	401	AUC_24_/MIC Cmax/MIC C_BP_ T_>MIC_

*The data reported in this Table have been collected using Ovid Medline^® ^with the following keywords: concentration-dependent; time-dependent; antibiotic; antimicrobial; PK/PD; breakpoint; efficacy.

Some antibacterial agents such as glycopeptides and some beta-lactams are referred to as being co-dependent.

This paper is organized as follows: In the Methods Section, we propose a logical extension of the known efficacy function in order to define the efficiency of a PK profile. In the Application and Results Section, we discuss some useful properties of our new approach and apply it to the particular case of variable drug intake. Finally, we give a general discussion to position our approach and findings within the current status of the field.

## Methods

### Weighted AUC: a rational parameter for assessing PK/PD efficiency

As mentioned in the background, recently introduced PK/PD-based breakpoint estimation was put forward to overcome drawbacks of threshold criteria, namely MIC, which determines *in vitro *antimicrobial efficacy. However, these PK/PD-based methods use drug exposure mainly through the AUC value (the amount of drug absorbed), whereas the variability in drug concentration time course is not integrated. This variability turns out to be an important factor in drug efficacy, as widely reported [27]. In bioequivalence studies, for example, it is common to combine AUC and Cmax to compare PK profiles and thus indirectly assess the expected drug efficacy. Therefore, to rely solely on the use of these PK parameters may not be sufficient for drawing reliable conclusions on drug efficacy. To employ these parameters efficiently and optimize their use for specific purposes, we need to adapt them by adding more information on drug PK/PD properties.

AUC-based drug efficacy is generally assessed through statistical methods such as scatter plots. Since PK/PD properties are not fully exploited, the relationship between drug efficacy and PK parameters only partially reflects the pharmacological properties. If additional PK/PD properties can be accounted for, the capacity of the actual empirical PK/PD-based breakpoint estimation is likely to be improved. Ideally, when a PK/PD relationship can be determined *in vivo*, the power of drug efficacy prediction can be maximized. However, exact dose-response relationships under *in vivo *situations are not easily accessible. This is the main restriction that prevents full exploration of drug efficacy prediction. Alternatively, combining the *in vitro *efficacy function (*E*) – the PK/PD relationship measurable in the laboratory – with AUC provides a better relationship (being more information-loaded) than that of drug efficacy in terms of AUC. As we will see, this combination can be considered an extension of the definition of AUC, thus relating to specific information on drug response.

In the case of antibiotics, dose-response or concentration-response curves against a microbial agent, also called killing or growth inhibition curves, can more easily be established under *in vitro *conditions. Several functions, such as linear, sigmoid or logistic, can be used to describe drug efficacy [28-31]. For example, consider drug efficacy *E *as a probability function expressing inhibition of bacterial growth in response to antibiotic concentrations. It can be modeled as:

(1)

where Emax is the maximum effect (normalized to one in this paper), EC_50 _the drug concentration that attains 50% of Emax, and H is the Hill constant [31]. Since this efficacy function carries rich information about the response in terms of concentration, it should and could be translated under *in vivo *conditions. In fact, the *in vivo *situation can be considered as a composite of many "local" *in vitro *cases. "Locally *in vitro*" here means that once the antibiotic reaches a certain site in the body (a target organ for example), it behaves in a similar way as *in vitro*. In the following, we will include this efficacy function *E *in our approach to predicting the drug's therapeutic performance and apply it to the case of concentration-dependent antibiotics.

To evaluate the performance of a PK profile, we chose to measure it by the expression efficiency, *Eff*, defined as follows:

(2)

where again *E *is a function related to drug efficacy, T is the therapeutic duration used as a reference period and n = 0, 1, ...

Compared to AUC, *E *here plays the role of a weighting function. We use it to include the information on the PK/PD relationship as an integral part of drug efficiency measurement expressed through *Eff*. This information can always be updated and integrated for this purpose. For the particular case of *E *= 1 and n = 1, we obtain the usual AUC definition, thus making our newly introduced efficiency function a direct extension of AUC.

**Figure 1:**
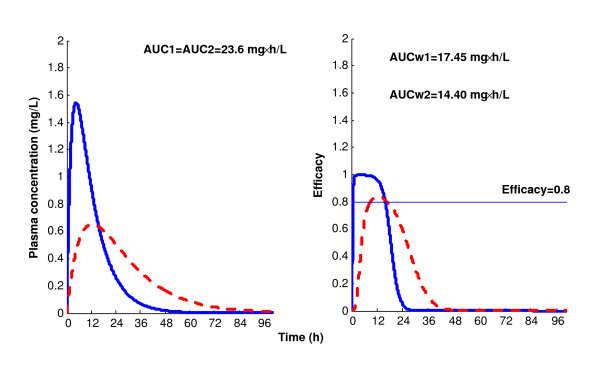
**The left panel depicts two PK profiles with the same AUC (23.6 mg × h/L)**. The solid curve illustrates rapid absorption while the dashed curve corresponds to slower absorption. The right panel depicts the corresponding efficacy vs time curves, which still show the difference in the PK profiles of the left panel; this difference is translated into the values of the corresponding efficiency AUC_W _(17.45 vs 14.40 mg × h/L). The efficacy of the high absorption regime lasts almost throughout the therapeutic period (24 h) beyond the target efficacy of 0.8 mg.h/L, while the lower absorption regime barely reaches this target.

As an illustration, we will show how the newly introduced efficiency function can differentiate between PK profiles with the same AUC. In Figure 1, the two PK profiles share the same AUC but noticeably different AUC_W_. In fact, this additional information level allows drug evaluation and assessment of therapeutic performance to be refined.

### Concentration-dependent antibiotics: weighted AUC method for antimicrobial efficiency

As mentioned, the effects of concentration-dependent antimicrobial agents are known to be proportional to concentration. Their efficacy is generally assessed through pharmacokinetic parameters, namely AUC or Cmax. To characterize the efficiency of concentration-dependent drugs, we propose to use the first order version of the efficiency *Eff*:

(3)

We notice that *Eff*_1 _contains information related to both AUC and concentration variation levels. In this newly proposed formula, these two elements are well integrated to reflect their contributions to the evaluation of drug performance. *Eff*_1 _can thus be considered an extension of the classical approach [14]. We refer to *Eff*_1 _as the weighted AUC and denote it by AUC_W_.

### Time-dependent antibiotics: an analytic expression for total antimicrobial efficiency

**Figure 2:**
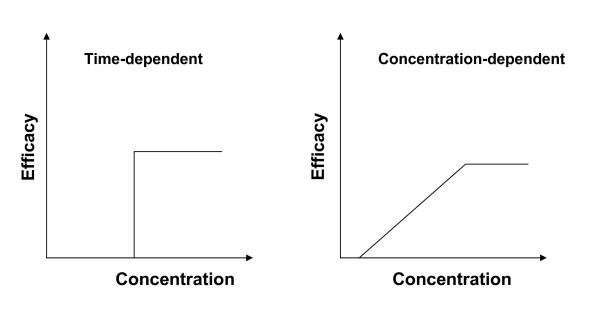
**Illustration of efficacy vs. concentration of the two groups of antimicrobial agents**. The time-dependent microbial agent in the left panel has an efficiency of all or none, i.e. there is a threshold concentration above which the drug is considered to be fully effective, and below which it is non-effective. The performance of the concentration-dependent antimicrobial agent in the right panel is known to be proportional to concentration.

The efficacy of a time-dependent drug depends on the percentage of time during which the concentration exceeds a specific value C_BP_, generally called the breakpoint. C_BP _acts as a threshold value: the drug is considered to be fully effective when its concentration is over this value, but non-effective otherwise (Figure 2). For time-dependent drugs, we formulate the efficiency as:

(4)

where *E *= χ is the indicative function. We recall here that the indicative function χ_A _of a set A is defined as: χ_A _(t) = 1 if t belongs to A; 0 otherwise. Hence, expressed in this way, χ will be 1 if C(t) > C_BP _and will be 0 otherwise. We notice that *Eff*_0 _is simply the cumulative time during which C(t) remains above the specific concentration value C_BP_, which turns out to be exactly the same classic definition for evaluating time-dependent efficacy. However, expressing it in this way, with explicit reference to the zero-order general efficiency *Eff*_*n *_function proposed above, helps us to understand the direct relationship of efficiency for different drug groups.

## Application and results

In the following, we will focus on concentration-dependent antibiotics to illustrate how the newly introduced weighted AUC method can be used.

### Efficiency equivalence between *in vivo *and *in vitro*

In pharmacology, estimation of drug efficacy is important for optimizing a drug regimen such that the best therapeutic outcome can be achieved. Generally, this estimation should be performed under *in vivo *conditions. Since drug concentrations within the body are unavoidably variable, and *in vivo*-induced randomness may also be superposed, *in vivo *estimation of drug efficacy is a complex problem. Microbiologists use *in vitro*-based methods for estimating antibiotic efficacy. These well-controlled *in vitro *studies can result in useful partial predictors for the *in vivo *potency of drug-microorganism interactions. Very often, efficacy-drug concentration curves are well established *in vitro*. This information makes it possible to establish a certain rule for efficacy equivalence between different real PK profiles such that the efficacy of a drug regimen can be objectively judged. Based on the efficiency function introduced above, two PK profiles are ascertained as **efficiency-equivalent**, i.e.*PK*_1 _⇔ *PK*_2 _*in efficiency*, if and only if they verify the condition:

(5)

More precisely, for concentration-dependent drugs, we can try to find a corresponding *in vitro *(constant) equivalent concentration C^e ^that is likely to produce the same efficiency provided by a given PK profile. In this case, for a given PK profile C(t), the corresponding equivalent *in vitro *concentration C^e ^is the solution of the equation:

(6)

For time-dependent drugs, the situation is different. The efficiency is the percentage of time during which the concentration remains above a specific value C_BP_. As *in vitro *concentrations can only have binary efficiency, we have to determine the threshold of time percentage for an effective drug regime. The efficiency of a PK profile can be compared with this threshold to measure its efficacy.

### Weighted AUC method and irregular drug intake

As an application of the AUC_W _method, we will consider the case of variability in PK profile generated by irregular drug intake. It is common sense that a deviation between real drug intake and the ideal prescribed dosing regimen is likely to have an impact on the pharmacokinetic profile and eventually the drug response. Non-compliance characteristics can be translated into some derived PK/PD parameters and pharmacological indices.

In a previous study, we investigated the impact of animal feeding behaviour on the pharmacokinetics of chlortetracycline (CTC), a widely-used antibiotic usually given through animal feed [32]. We modeled a widely reported animal feeding behaviour and associated it with the CTC disposition model to obtain a feeding behaviour-PK (FBPK) model. Using this model, we revealed the PK variability induced by random drug intake and assessed its main characteristics [32].

In the present paper, we will focus on the estimation of efficiency of CTC in this particular context of irregular drug intake. We have to mention that similar reasoning and analysis can be accomplished using other sources of variability impacting pharmacokinetics. Since CTC is a concentration-dependent antibiotic widely used in collective medical therapy, we will base our analysis on the method we propose for this antibiotic class. For the purpose of illustration, we will use the individual FBPK we previously developed [32]. The case of an animal population can be developed by adding the inter-variability to the associated PK parameters. In the following, we will answer the following questions: Can the "efficacy performance" of PK profile be characterized uniquely by its average concentration value? What is the extent of *in vitro *equivalent concentrations that an average concentration can reach? Since we only have access to the drug concentration in feed, what can we say about the potential efficacy of various drug concentrations in feed compared to that of MIC?

### An advanced PK model integrating swine feeding behaviour: an FBPK model

In veterinary medicine, the problem of optimal use can arise for drugs administered through feed, a widely-used practice for therapeutic, metaphylactic or prophylactic treatment of bacterial infections [33]. As a consequence, animal feeding behaviour directly influences systemic exposure to drugs. However, variation in the feeding behaviour of animals medicated through feed has been overlooked for more than 50 years, during which feed antibiotic therapy remained empirical. Using widely-reported descriptions of swine feeding behaviour, we have mathematically formulated and integrated this behaviour model into a PK model (FBPK) in order to analyze its influence on systemic exposure to drugs quantitatively [32]. We include here a brief review of the FBPK model. Complete details about the model and its analysis can be found in [32].

The feeding behaviour model consists of two typical daily feeding activities: routine peak periods complemented by inter-peak periods of free access to feed. The routine peak periods correspond to intense feeding activities generally referred to as morning and afternoon peaks. Meals consumed between peak periods are referred to as inter-peak meals. The time intervals between two successive inter-peak meals are reported to follow a Weibull distribution. Since the animal consumes the feed in a quasi-continuous manner during the peak periods, and considering the low elimination rate of CTC, we have modeled the feeding activity during these periods as an infusion process, which gives rise to the following concentration time-course:

(7)

(8)

where [T_s_, T_e_] is the duration of the peak period, DOSE is the drug concentration mixed in the feed, with units in ppm,  is the average ingestion rate, F is the bioavailability, K_a _and K_e _are the absorption and elimination rates respectively, and V is the volume of distribution.

**Figure 3:**
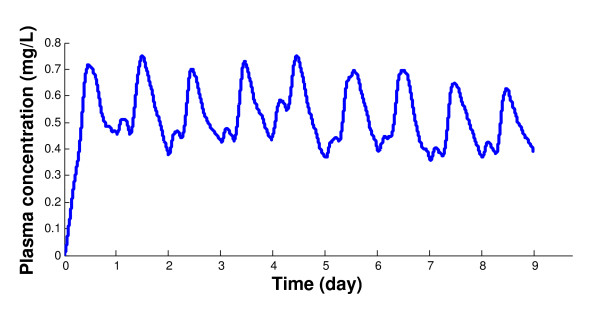
**A typical plasma drug concentration under conditions of irregular drug intake, with DOSE = 500 ppm CTC mixed in the animal feed**.

Inter-peak meals are modeled as individual boluses entering the gastrointestinal tract because their durations are relative short compared to the inter-meal intervals. A two-parameter Weibull distribution is used to account for these irregular feeding events of free access to feed. Figure 3 illustrates a typical PK profile of an animal receiving 500 ppm of drug mixed through feed.

### Estimation of MIC breakpoints in animal populations

By definition, MIC breakpoints refer to critical drug concentrations that characterize specific antibacterial activities. The values of these MIC breakpoints are highly pertinent to the pharmacokinetic properties as well as to the pharmacodynamic killing capacities of these drugs with respect to particular bacterial strains. In the clinical setting, MIC is considered an important reference index in choosing effective dose regimens. However, because of the evident large variation in concentration time course and the unavoidable pharmacokinetic variability under the *in vivo *situation, the true PK/PD relationship is generally more complex. Using a single static value of MIC for the decision process is dubious or even misleading. Therefore we have to take account of dynamic *in vivo *properties when estimating drug efficacy.

In the following, we will use the above-developed feeding behaviour-PK model to show how one can obtain breakpoint information, and of what kind, for an *in vivo *situation.

To do this, we adopt a Monte Carlo approach to generate, for an animal X, possible drug inputs prior to drug disposition. The corresponding concentration time courses are then produced with these drug inputs. To explain our approach, we need to introduce some new concepts and their notations.

• DOSE: drug concentration mixed in feed, with units of ppm.

• : average over a time duration T of one concentration time course generated by Monte Carlo; it is AUC-based.

• : global mean of all average concentrations .

• : 95% higher mean concentration where 95% of  are below this concentration.

• : 95% lower mean concentration where 95% of  are above this concentration.

• : *in vitro *equivalent concentration (Eq. 2) of C_i_(t), where 0 ≤ t ≤ T; it is AUC_W_-based.

• : global mean of all *in vitro *equivalent concentrations .

• 95% higher in vitro equivalent concentrations , where 95% of  are below this concentration

• : 95% lower *in vitro *equivalent concentrations , where 95% of  are above this concentration.

**Figure 4:**
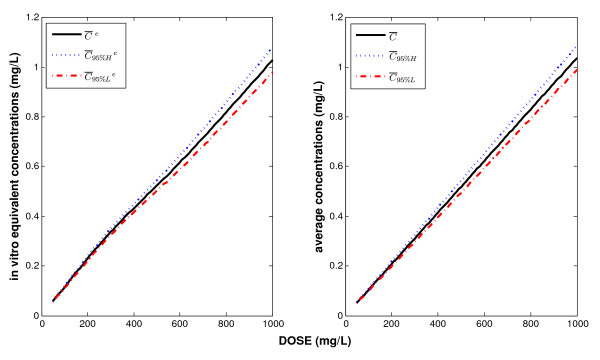
**The left panel shows the *in vitro *equivalent concentrations versus DOSE; the solid, dotted and dash-dot lines are , ,  respectively**. The right panel shows the average concentrations versus DOSE; the solid, dotted and dash-dot lines are , ,  respectively.

Using the FBPK model, we can estimate the above concentrations versus DOSE (Figure 4). This figure shows the 95% confidence intervals of *in vitro *equivalent concentrations  and average concentrations in terms of DOSE. For example, given a DOSE = 400 ppm, we obtain [, ] = [0.417 mg/L, 0.450 mg/L] and

[, ] = [0.397 mg/L, 0.435 mg/L].

We can consider that a DOSE is **at least 95% efficiency-equivalent **to an *in vitro *concentration C_eff _by defining 95% of equivalent *in vitro *concentrations generated by this DOSE as being above *C*_*eff*_. In our case, for a given DOSE, we have the relationship *C*_*eff *_=  (*Dose*) according to this 95% efficiency-equivalence criterion.

However, with each DOSE, we can also associate a 95% confidence interval of average concentrations represented by [ (*Dose*),  (*Dose*)] as illustrated in the right panel of Figure 4.

**Figure 5:**
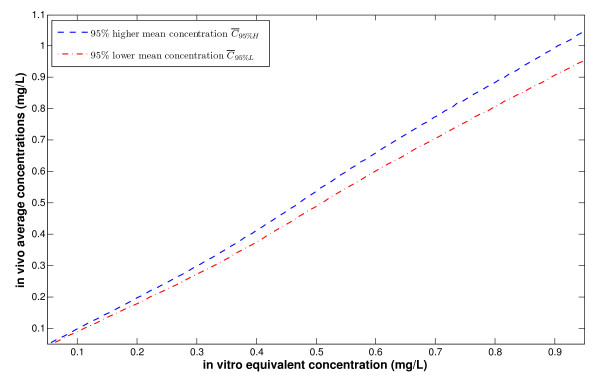
***In vivo *mean concentrations versus *in vitro *equivalent concentrations**.

Then for each **at least 95% efficiency-equivalent ***in vitro *concentration , it corresponds an interval of average concentrations [, ]. This clearly indicates that under *in vivo *situations, we have an associated uncertainty in average concentrations that may correspond to the same specific PK efficiency value. In other words, the *in vivo *average concentration when used as a breakpoint to indicate the efficacy of a dosing regimen can only be interpreted probabilistically. This result is reported in Figure 5.

To a given target value Ce (*in vitro *target), there corresponds a DOSE that gives an interval of equivalent concentrations (hence equivalent efficacy) lying above Ce.

**Figure 6:**
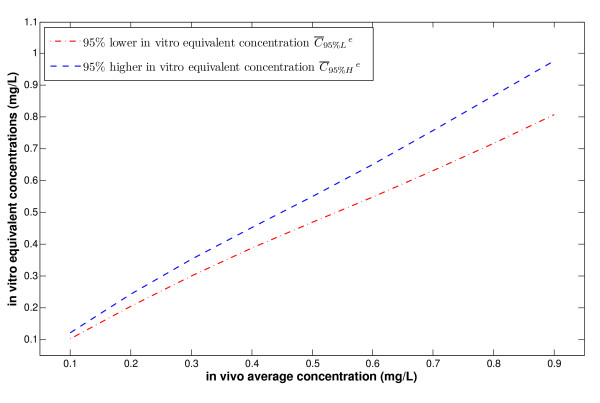
***In vitro *equivalent concentrations versus *in vivo *average concentrations**.

However, a given average concentration , which is in fact measured theoretically (using AUC for example), may be the result of many different DOSEs. We can write this corresponding interval as [DOSElow, DOSEhigh] as a function of . For DOSElow, the lowest *in vitro *equivalent concentration that can be attained by  in the sense of 95% probability will be given by  (*DOSElow*). The same applies to DOSEhigh, where the highest *in vitro *equivalent concentration that can be attained by  in the sense of 95% probability is given by  (*DOSEhigh*). Hence, for each , there is a corresponding whole interval of possible *in vitro *equivalent concentrations given by these two extreme values and denoted by [ (*DOSElow*),  (*DOSEhigh*)]. This result is reported in Figure 6. The illustrated (one-to-one) relationship between  and DOSE highlights the possibility (need) to dissociate between the average concentration and efficacy, thus questioning the general practice of evaluating efficacy through average concentrations.

**Figure 7:**
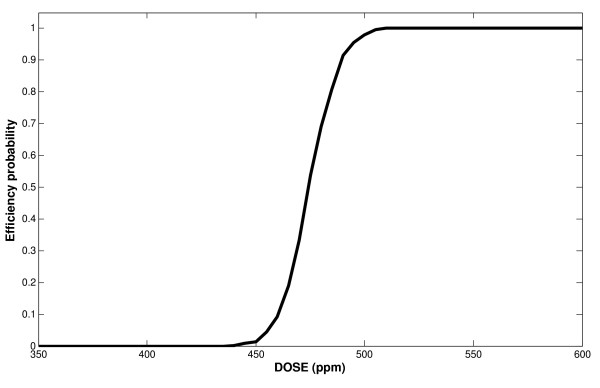
**Therapeutic success probability across DOSEs**. MIC = 0.5 mg/L.

To answer the third question, we consider a MIC = 0.5 mg/L, which is the breakpoint normally used in practice for the evaluation of CTC efficacy. For different values of DOSE, we estimate the probability of the *in vitro *equivalent concentrations with values above MIC. A plot of these probabilities versus DOSE is given in Figure 7. We can see that for low DOSE values, it is almost certain that the therapy is non-efficient while the opposite is the case for high DOSE where success is almost secured. However, there is a critical zone of drug concentration in feed (DOSE) within which a given DOSE has a certain potential of success or failure.

### Robustness of weighted AUC approach

**Figure 8:**
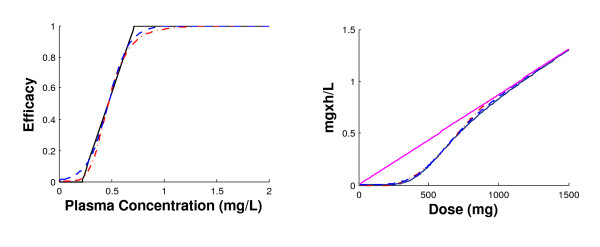
**Robustness of weighted AUC method**. Left panel represents the three most widely used efficacy functions *E*: solid line for the linear function, broken line for the Emax function and dotted line for the logistic function. The corresponding AUC_W _are plotted in the right panel with respect to concentration, along with AUC (solid thick line). The difference in AUC_W _value estimated for these three efficacy functions is negligible compared to the differences from AUC.

Here, we will explain and illustrate some advantageous properties of AUC_W _compared to AUC. In its integration formula, the AUC_W _method incorporates the *in vitro *efficacy function *E*, thus penalising lower drug concentrations in an appropriate way. Hence, AUC_W _constitutes an improvement over AUC since the nonlinearity principle in drug efficiency is respected (Figure 8, right panel). Also, AUC_W _proves to be robust in terms of the efficacy function *E*, which represents an important feature when it comes to application. Indeed, we have generated AUC_W _for three efficacy functions, namely the linear, Emax and logistic functions. These functions along with the corresponding AUC_W _are plotted in Figure 8, left and right panels respectively. For sake of comparison, the AUC is also depicted on the right panel. This figure shows that the difference between AUC and AUC_W _is more noticeable than that of the three generated AUC_W_s.

## Discussion

Unlike the ideal *in vitro *conditions, where major guidelines for drug efficacy are routinely established for stable drug concentrations, it is natural that high variability arises *in vivo *and thus raises concerns about the applicability of *in vitro*-established principles. This *in vivo *variability may have various origins and forms [34,35]. One of these sources is structural and is directly linked to drug disposition and elimination processes (generally referred to by ADME: Absorption, Distribution, Metabolism and Elimination), where the drug concentration time course is often described using ordinary differential equations. These ADME scenario components are generally mimicked, separately, under laboratory conditions but hardly synthesized as a whole. The well known PK parameters such as AUC and Cmax are specifically designed to reflect this drug exposure variation in the PK/PD association. Beyond this structural variability, other pharmacokinetic variability is widely recognized and turns out to be an important influence on drug efficacy. Neglecting variability when assessing therapeutic efficacy may lead to wrong conclusions [32,35-37]. In the current article, we have shown how, instead of relying solely on AUC or other single parameters, the entire (*in vitro *or *in vivo*) pharmacodynamic function should be considered in a more integrated way for evaluating and developing antibiotic treatment protocols. Being concerned with this issue, we have directly generalized the classical AUC-based methods and rendered drug evaluation more efficient by including richer information on the PK profile.

As a static efficacy-threshold parameter widely used for breakpoint assessment, MIC does not include drug disposition or other potential variability information. In fact, MIC is measured under almost deterministic conditions since variability is likely to be smaller *in vitro *than *in vivo*. However, antibacterial efficacy is the result of a complex dynamic process that depends on concentration and time. Hence, relying on such *in vitro *values may be risky since real *in viv*o values can spread over a relatively large range. Generally, these *in vitro *values are used to refer to mean *in vivo *values. However, we have seen here that using the average concentration as a reference value can lead to ambiguous interpretation of drug efficacy since various PK profiles are likely to share the same average concentration while having different therapeutic performances. Under *in vivo *conditions, all these parameters should be reconsidered and adapted to reflect this varying situation. In this context, it is thus common sense to have recourse to a probabilistic approach, as we illustrated in the examples above.

Another interesting issue arising directly from our method concerns bacterial antibiotic resistance. It is known that under-exposure of bacterial strains to antibiotics is the main cause of resistance. When traditional exposure indices such as AUC or Cmax are used to evaluate drug efficacy, the prediction is linearly related to antibiotic exposure. Since these derived indices are proportional to dose, the real mechanism of drug killing is not incorporated as the linear property remains unchanged when either drug exposure or dose is used. In some recent work, a trend in this direction can be noticed [28,38-40]. Using our efficiency evaluation approach, we observe that for low doses the traditional AUC-based method gives an optimistic efficacy evaluation as the drug killing properties are ignored in its expression form. However, when we account for killing properties through the efficacy curve as we did in our efficiency formula, we clearly see that the drug efficacy evolves more slowly than the corresponding dose. In our example, under a 500 ppm DOSE, the drug efficiency estimated using our method is half that of the AUC-based method. Hence, for lower doses, there is a good chance of being in low efficiency situations where the risk of antibiotic resistance is higher than can be assessed using traditional methods. These results suggest that further investigation in this direction is needed, especially because lower doses are usually related to irregular drug intake, such as drug holidays or cases of antibiotic abuse. We believe that more advanced methods should be developed to address this problem. Our approach is one step towards this end. We propose here a logical way of evaluating drug efficiency on the basis of *in vitro *efficacy information and the PK profile. This can be relevant to antibiotic development, especially for the estimation of the efficacy dose in phase II. In this work, we have used killing curves to illustrate our methods. This does not prevent extension of our approach to other antibacterial drugs. In fact, we have already suggested a possible approach for time-dependent drugs. However, for other complex types of effect, there is a need for further investigation of the mechanism and for embedding the results in the efficiency form that we proposed. In fact, the complex facets of antibacterial activities are not limited to this simple classification. For some antibiotics, such as glycopeptides, a combination of concentration and time of exposure may both be relevant [7,26]. The coexistence of bactericidal and bacteriostatic properties in these drugs makes them co-dependent on both concentration and time. Nonetheless, the classification into concentration-dependent and time-dependent suggested by the ISAP for antibiotics provides an objective basis for judging antibiotic performance. It is interesting to note that these two patterns fall within the two extreme cases of antibiotic efficacy, as illustrated by the curves of Figure 2[28].

Finally, owing to the complexity of biological systems – the human body here, as well as the mechanisms involved in the bacteria killing capacities of drugs – new methods are being developed every day. Some of them use very sophisticated theories that include every known facet of mechanism; others seek to ignore these complexities and use elementary mathematics with an empirical philosophy. The former struggle with the applicability of their methods, while the latter often lack logistic links to the underlying mechanism. A trade-off should be found to balance these tendencies. We think that our article may inspire progress on this path.

## Conclusion

In this paper, we have proposed a logical generalisation of the classical AUC method by introducing the "efficiency" of a PK profile, which involves the efficacy function as a weight. We have formulated these methods for both classes of concentration-dependent and time-dependent antibiotics. We have illustrated the approach developed using the particular case of variable drug intake. We have also shown how the new approach can overcome some limitations of the classical methods for assessing drug efficacy.

## Competing interests

The authors declare that they have no competing interests.

## Authors' contributions

GDG participated in the initiation and worked on the whole study including the results, outline, writing, and editing of the manuscript. The study was conducted under the main supervision of JL and FN, who were involved in the conception of this work, including the methodology and the writing of this paper for its intellectual content. All authors have read and approved the final manuscript.
